# Numerical research on disastrous mechanism of seepage instability of karst collapse column considering variable mass effect

**DOI:** 10.1038/s41598-024-63344-w

**Published:** 2024-06-17

**Authors:** Cao Zhengzheng, Zhang Shuaiyang, Li Zhenhua, Du Feng, Huang Cunhan, Wang Wenqiang

**Affiliations:** 1 International Joint Research Laboratory of Henan Province for Underground Space Development and Disaster Prevention, School of Civil Engineering, Henan Polytechnic University, Jiaozuo, 454000 Henan China; 2 Henan Mine Water Disaster Prevention and Control and Water Resources Utilization Engineering Technology Research Center, Henan Polytechnic University, Jiaozuo, 454000 Henan China; 3 Collaborative Innovation Center of Coal Work Safety and Clean High Efficiency Utilization, Jiaozuo, 454000 Henan China

**Keywords:** Collapse column, Water inrush, Disastrous mechanism, Seepage instability, Variable mass effect, Coal, Civil engineering

## Abstract

In order to reveal the disastrous mechanism of seepage instability of karst collapse column considering variable mass effect, a variable mass fluid–solid coupling mechanical model of water inrush is established, by considering the random distribution characteristics of a collapse column. Taking Qianjin coal mine as the research background, based on the Weibull distribution theory, the heterogeneous distribution characteristics of rock mass is described, and COMSOL Multiphysics numerical simulation software is employed to simulate the seepage characteristics and inrush water changes in collapse columns under different conditions of homogeneity, water pressure, and initial porosity. The research results show that the greater the homogeneity is, the more water conduction channels are formed, and the porosity increases accordingly, when considering the influence of different homogeneity on the seepage characteristics of broken rock mass, which eventually leads to water inrush accidents and a sharp increase in water inflow. Besides, when studying the seepage evolution law of different water pressures on a broken rock mass, an elevation of water pressure dramatically increases the porosity and seepage rate of the water. Over time, the broken rock particles gradually migrate and the fine particles are transported and eroded by the water flow, resulting in changes in the seepage characteristics and the formation of potential water diversion channels. Finally, when taking into account the effect of different initial porosity on the fractured rock mass seepage characteristics, the greater the original porosity is, the higher the seepage velocity is, and the particle migration increases the permeability. This leads to a more pronounced conductive water passage formation, which reveals the disastrous mechanism of seepage instability of karst collapse column considering variable mass effect.

## Introduction

Karst collapse column is a naturally formed geological structures and is one of the disasters that seriously threaten the safety aspect of coal mine production. Ground karst landforms have ecological problems, such as scarce soil, scarce surface streams, and a rugged terrain^1,2^. Ground karst landforms include sinkholes, karst buckets, karst springs, etc. Atmospheric precipitation seepage into karst caves and underground rivers through karst structures, and a large amount of karst water is stored in rock layers, providing a sufficient water supply. Under mining-disturbance conditions, the collapse column easily connects to natural aquifers, forming a smooth water channel^3^. The filling material of the collapse column migrates and loosens with the water flow, affecting the normal mining conditions in coal mines and leading to water inrush disaster^4,5^.

Yin Shangxian et al.^6^ established a “thick-walled cylinder” mechanical model for water inrush in collapse columns and applied the shear damage theory method of structural analysis to derive the theoretical model of water inrush of collapse column. Pradipkumar et al.^7^ and Cherubini et al.^8^ carried out steady-state permeability tests on fractured rock masses, which shows that seepage in fractured rock masses generally obey the Forchheimer equation rather than Darcy’s law. Based on the water inrush mechanical model of collapse column, Tang et al.^9^ used the medium-thickness plate theory and the yield damage criterion to obtain the level of the crucial water pressure concerning hidden collapse column water inrush in a floor plate. In this study of fluid–solid coupling effect, Terzaghi^10^ studied the fluid–solid interaction and proposed the concept of “effective stress” for the first time, which was widely used in soil mechanics studies. Javadi et al.^11^ established a geometric model based on CFD, and the flow law of the fluid in the fracture was described by using a polynomial similar to the Forchheimer equation. Wu Yongping et al.^12^ studied the failure mechanism of the surrounding rock of collapse columns by considering the fluid–solid coupling effect. Yao et al.^13^ studied the water inrush process of collapse column under the action of filling particle loss by considering the in-homogeneity of the fractured rock mass of the collapse column, and obtained the water inrush mechanism of collapse column under the action of stress–seepage coupling. Zhang Hongmei et al.^14^ analyzed the damage characteristics of collapse columns under the action of stress–seepage coupling. Basak et al.^15^ and Tartakovsky et al.^16^ discovered the existence of the high-speed non-Darcy flow when they observed the problem of flow movement in confined aquifers.

In terms of the numerical simulation of water inrush in collapse column, previous researchers have employed various numerical calculation software to calculate and simulate the evolution characteristics of the seepage field and the entire process of water inrush of collapse column under different mining conditions. Zhang Kai et al.^17^ utilized Comsol Multiphysics numerical simulation software to obtain the variations of porosity and permeability velocity in fractured rock mass of a collapse column in different time periods. Zhu et al.^18^ adopted Comsol Multiphysics programming to develop the intrinsic relationship of the rock breakage process under the fluid–solid coupling condition, and developed an ontological relationship for the rock damage process under the condition of fluid–solid coupling. Liu Zhijun et al.^19^ utilized the ANSYS finite element software to study the water inrush of collapse column, and investigated the distribution of strain, hydraulic pressure, and stress in the floor of coal seam. Yin Shangxian et al.^20^ applied FLAC3D numerical simulation software to analyze the mechanism of water inrush of collapse column. Shi Wenhao et al.^21^ applied FEPG finite element software to create the Fortran source program and calculated the non-Darcy flow model of the broken rock mass, which simulated the transient flow of water inrush. The numerical calculations were carried out to simulate the whole process of water transient flow in the fractured rock mass. Yang Tianhong et al.^22^ and Huo Bingjie et al.^23^ used COMSOL Multiphysics numerical software to propose the principle of the three flow regime transitions of water inrush in collapse column.

For the prevention of water inrush disasters, Ma Tianxing^24^ developed an normal cloud model for predicting the risk of water inrush in the floor plate of coal seam. Ma Lianjing^25^ constructed a water damage prevention and control method of hydrophobic depressurization, using water discharge test and numerical modelling of groundwater flow. Wang Wenqiang et al.^26^ proposed an mining method to control roof water inrush, which provided a reliable basis for preventing and controlling roof water inrush disasters. Hoang, UT et al.^27^ adopted the super-quadratic discrete element method to systematically study the influence of particle shape on particle collapse, and further enhanced understanding of the unique influence of particle shape. Wang et al.^28,29^ conducted the application of liquid nitrogen cooling treatment of granite, and proposed that this method can effectively weaken the mechanical properties of the rock layers. Cao et al.^30^ established a numerical simulation of grout diffusion in single slab crack sand, and analyzed the diffusion law of grouting slurry in cracks with different rheological and consistency indices. In the detection of collapse column, the three-dimensional seismic method is the most commonly used technique. Many scholars summarized the latest acquisition method technologies and observation system design methods, which provided a comprehensive and systematic introduction to the design and construction of three-dimensional seismic observation system on land^31–33^.

For the research on water inrush of collapse column, seepage change is identified as the root cause of water inrush disaster. Most prior researches are focused on the perspective of structural damage to study the changes in rock seepage characteristics, and the significant impact of water erosion on the permeability characteristics of broken rocks in collapse column is overlooked. Therefore, this paper adopts the perspective of the variable mass fluid–solid coupling effect to study the disastrous mechanism of seepage instability of karst collapse column, in order to unveil the mechanisms of water inrush of collapse column.

## Establishment of mechanical model

### Basic hypothesis

To establish a mechanical model of water inrush in collapse column and the evolution of fluid particle loss, the following assumptions are proposed: the motion velocity of suspended particles is approximately equal to the fluid velocity; the influence of particles in the fluid on the fluid permeability characteristics is neglected.

### Particle mass conservation equation

The motion equation of particles is^34,35^

1 $$ \frac{\partial }{\partial t}\left[ {\varphi \left( {1 - c} \right) + \nabla \cdot \left[ {\varphi \left( {1 - c} \right)q_{f} } \right]} \right] = 0 $$

In the above Eq. (1), $\varphi$ is the porosity; *c* is the volume concentration of suspended particles; $q_{f}$ is the seepage velocity; $\vec{\nabla }{ = }\frac{\partial }{\partial r}\vec{e}_{r} + \frac{1}{r}\frac{\partial }{\partial \theta }\vec{e}_{\theta } + \frac{\partial }{\partial z}\vec{e}_{z}$ is the Hamiltonian operator.

### Seepage field equation

The Brinkman equation is an infiltration equation between Darcy flow and Navier–Stokes flow^36–38^. In COMSOL Multiphysics, this Brinkman equation with Forchheimer correction is used to describe fluid flow:

2 $$ q_{f} \left( {\frac{\eta }{k} + \beta_{f} \left| {q_{f} } \right| + \frac{{Q_{br} }}{\varphi }} \right) = \nabla \left\{ { - pI + \frac{\eta }{\varphi }\left[ {\nabla q_{f} + \left( {\nabla q_{f} } \right)^{T} } \right]} \right\} + F $$

3 $$ \rho_{l} \nabla \cdot \left( {q_{f} } \right) = Q_{br} $$

In the above equations, *k* is the infiltration rate; *p* is the fluid pressure; ${\rho }_{l}$ is the fluid density; ${Q}_{br }$ is the source sink term; $\eta$ is the dynamic viscosity; ${\beta }_{f}$ is the no-Darcy divisor; *F* is the the volume force affecting the fluid; *I* is the unit matrix.

### Porosity permeability relationship equation

The relationship between permeability and porosity of a fractured rock mass is described by the following equation:

4 $$ k = k_{0} \left( {\frac{\varphi }{{\varphi_{0} }}} \right)^{3} \left( {\frac{{1 - \varphi_{0} }}{1 - \varphi }} \right)^{2} $$

In the equation, ${k}_{0}$ is the initial permeability; $\varphi_{0}$ is the initial porosity.

### Heterogeneous theory of rock parameter

The distribution of natural micro-defects in rock masses leads to significant discontinuities, heterogeneity, and anisotropy in physical and mechanical properties. A collapse column represents a form of heterogeneous geological material. In numerical simulations, heterogeneity in rock masses is typically modeled by assigning varied physical and mechanical parameter values to the microscopic structural units. The distribution characteristics of heterogeneity in rock masses can be described by the WeiBull distribution. By utilizing Matlab to generate data series conforming to the WeiBull distribution, and then assigning these to the corresponding micro-elements of rock masses, the non-homogeneity within rock specimens can be characterized. Integrating the above equation yields a cumulative distribution function:

5 $$ f(x) = \frac{m}{n}\left( \frac{x}{n} \right)^{m - 1} \exp \left[ { - \left( \frac{x}{n} \right)^{m} } \right] $$

Given that *n* and *m* are constant, rational numbers can be derived using the inverse function,

6 $$ x = n( - \log (1 - F(n)))^{\frac{1}{m}} $$

WeiBull-distribution function expectation is:

7 $$ E(x) = n\Gamma \left( {1 + \frac{1}{m}} \right) $$

In the above equations presented above, *x* is the independent autonomous variable, representing the physical–mechanical parameters to be produced; *n* is the scale parameter; *m* is the shape parameter, indicative of representing the rock heterogeneity.

**Figure 1:**
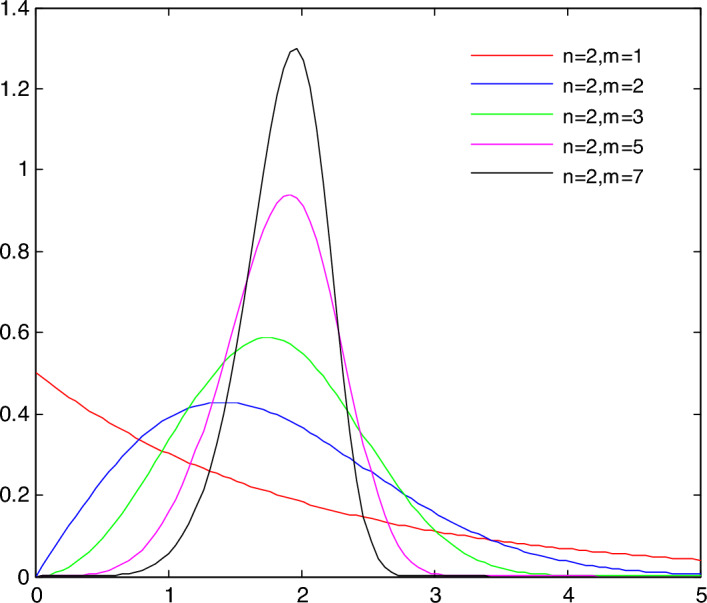
Weibull probability density function curve with different parameters.

The Weibull distribution function image is formed when *n* = 2 and *m* is 1, 2, 3, 5, and 7, as depicted in Fig. 1. The Weibull distribution simplifies to an exponential function when *m* is set to 1. When *m* is set to different values, the abscissa value corresponding to the curve highest point is near; the more random numbers are concentrated in the middle of the curve, the greater the shape parameter *m* is. The Weibull distribution effectively models the heterogeneity within rock masses, and a significant influencing component is the shape parameter *m*.

## Numerical model of water inrush in collapse column

Coal mining can induce water inrush in collapse column. The excavation in coal seam disrupts the equilibrium of surrounding rock and water, generating numerous random pores within the collapse column. Water seepage causes the particles in the pore space to be continuously brought out, resulting in the further expansion and penetration of the fissures. Erosion by the liquids leads to expansions of fine particles and penetrations fissures, ultimately forming a stable water-conducting channel.

### Establishment of a numerical calculation model

**Figure 2:**
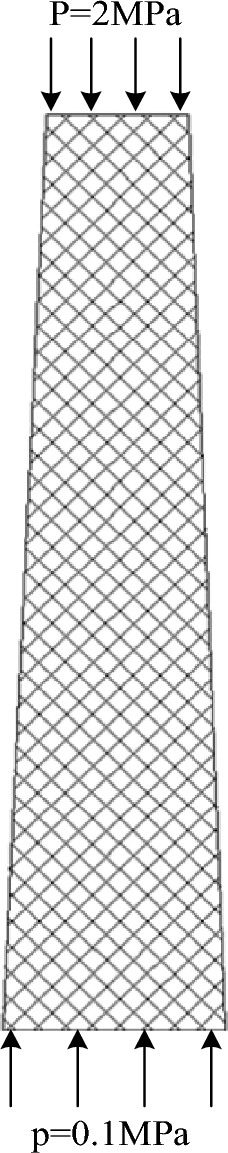
Numerical calculation model.

Figure 2 illustrates the cross-section of the model. The collapse column is characterized by a height of 185 m, with bottom and top diameters of 20 m and of 10 m. The seepage boundary conditions are as follows: the water pressure at the upper boundary is *p* = 2 MPa, the water outlet serves as the bottom boundary, with the pressure set to air pressure (*p* = 0.1 MPa). The left and right boundaries are impermeable. Monitoring time points are set at 5000 s, 10,000 s, 15,000 s, and 25,000 s. The surrounding rock of the broken rock mass is set to 0.6 MPa, with homogeneity *m* set to 3. Table 1 is the main parameters of the model.

**Table 1:** Values of major model parameters.

Physical parameter	Collapse column	Units
Rock mass density ${\rho }_{s}$	2260	Kg/m^3^
Elastic modulus of rock mass* E*	1.0	GPa
Poisson ratio *ν*	0.3	–
The dynamic viscosity of water $\mu$	1 × 10^−3^	(Pa·s)
Initial average porosity ${\varphi }_{0}$	0.1	–
Upper boundary pressure *P*	2 × 10^6^	Pa
Concentration *c*	0.01	–
Initial permeability* k*_*0*_	5 × 10^−12^	m^2^
Homogeneity *m*	2/3/5/7	–

### Numerical simulation calculation scheme

To investigate the impact of various variables on fluid flow in fractured rock formations, this study simulates the seepage characteristics of such formations under two distinct conditions. Table 2 provides details of the specific scenarios. Scenario 1 investigates the impact of varying initial pore homogeneity on water flow. Scenario 2 examines the impact of varying water pressures on the evolution of porosity and seepage velocity in the fractured rock mass. Scenario 3 explores the effect of varying initial porosity on water flow in the fractured rock mass.

**Table 2:** Numerical simulation schemes.

Option 1 the effect of homogeneity	Operating condition 1: *m* = 2
	Operating condition 2: *m* = 3
	Operating condition 3: *m* = 5
	Operating condition 4: *m* = 7
Option 2 the effect of different water pressures	Operating condition 1: *p* = 0.5 MPa
	Operating condition 2: *p* = 1 MPa
	Operating condition 3: *p* = 1.5 MPa
	Operating condition 4: *p* = 2 MPa
Option 3 the effect of initial porosity	Operating condition 1: $\varphi =0.05$
	Operating condition 2: $\varphi =0.075$
	Operating condition 3: $\varphi =0.15$
	Operating condition 4: $\varphi =0.175$

## Results analysis of numerical simulation

### The effect of different pore homogeneity on water inrush

**Figure 3:**
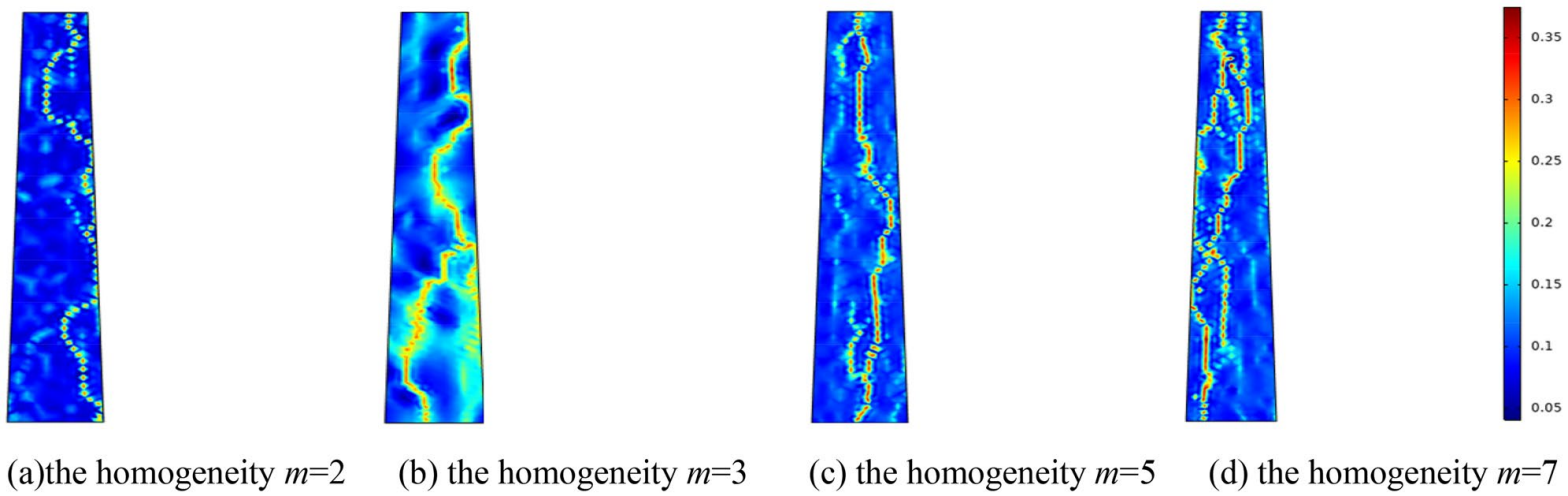
Porosity distribution with different homogeneity at the same time.

**Figure 4:**
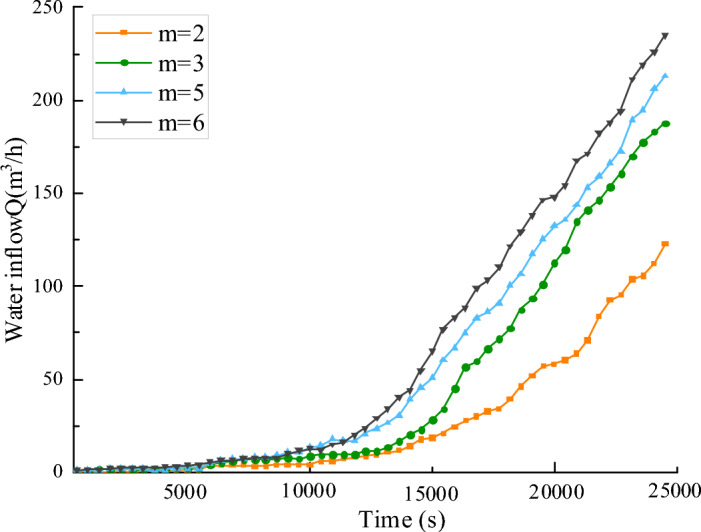
Relation curve of water inflow and time of broken rock mass under different homogeneity.

Considering the evolution law of infiltration in fractured rock mass under varying pore homogeneity conditions, the pore homogeneity is set to *m* = 2, *m* = 3, *m* = 5, and *m* = 7, respectively. Setting the surrounding rock pressure of the fractured rock mass at 0.5 MPa, the water pressure at 2 MPa, and the initial porosity at 0.1. Figure 3 illustrates the variation in porosity of the fractured rock mass at time *t* = 25,000 s under various pore homogeneity conditions. From the porosity distribution cloud map in Fig. 3, the configuration of the water channel is intricately linked to the pore homogeneity of the fractured rock mass. Specifically, when the *m* value is small, the formed water channel is single; when the homogeneity *m* is high, multiple water channel is formed, leading to an increase in water inrush. Figure 4 illustrates the variation curve of water inflow in collapse columns under various conditions of pore homogeneity in fractured rock masses. From the water inrush curve in Fig. 4, it can be seen that after 15,000 s, the water inflow of the collapse column suddenly increases, culminating in a water inrush disaster. Applying grouting reinforcement to the collapse column before this critical juncture can prevent water inrush accidents.

### Impact of varying water pressures on the permeability properties of confined columns

#### Porosity evolution over time under varying water pressures

**Figure 5:**
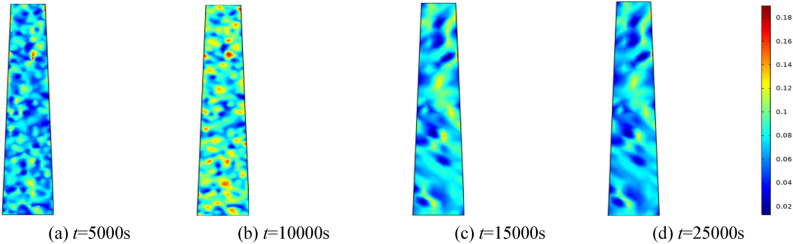
When the water pressure *p* = 0.5 MPa, the porosity distribution at different times.

**Figure 6:**
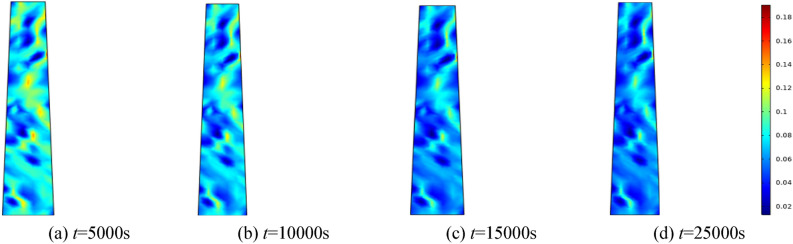
When the water pressure *p* = 1 MPa, the porosity distribution at different times.

**Figure 7:**
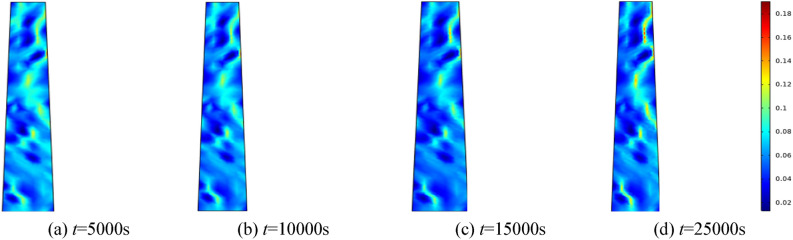
When the water pressure *p* = 1.5 MPa, the porosity distribution at different times.

**Figure 8:**
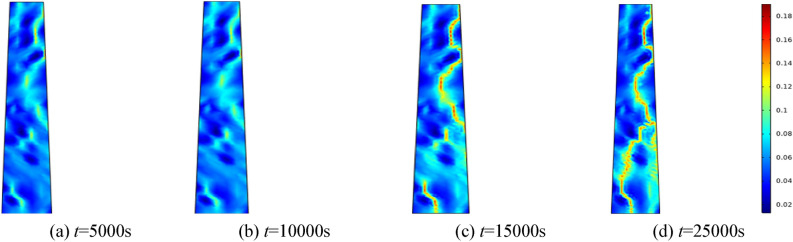
When the water pressure *p* = 2 MPa, the porosity distribution at different times.

To investigate the effect of water pressure on the percolation characteristics of the fractured rock mass, water pressures of 0.5 MPa, 1.0 MPa, 1.5 MPa, and 2.0 MPa are applied, respectively. The homogeneity parameter *m* is set to 3, and the surrounding rock of the fractured rock mass is established at 0.5 MPa. Selecting 5000 s, 10,000 s, 15,000 s, and 25,000 s for analysis. Figures 5, 6, 7, 8 correspond to the distribution cloud maps of the model porosity at different times points under water pressures of 0.5 MPa, 1 MPa, 1.5 MPa, and 2 MPa, respectively.

By analyzing porosity under varying water pressures, it is evident that as water pressure increases, porosity increases more rapidly and the formation of water-conducting channels becomes more pronounced. At the initial stage, the porosity distribution is random. As seepage time increases, the porosity value also ascends. Water seepage and erosion cause broken rock particles to migrate, damaging the internal pore and skeleton structure. Fine particles are transported by the water flow, leading to rapid porosity increase in some areas, while changes in other areas manifest more gradually. The occurrence of water inrush disaster can be attributed to the gradual expansion of the seepage fissure connection, which eventually formed a water conduction channel.

**Figure 9:**
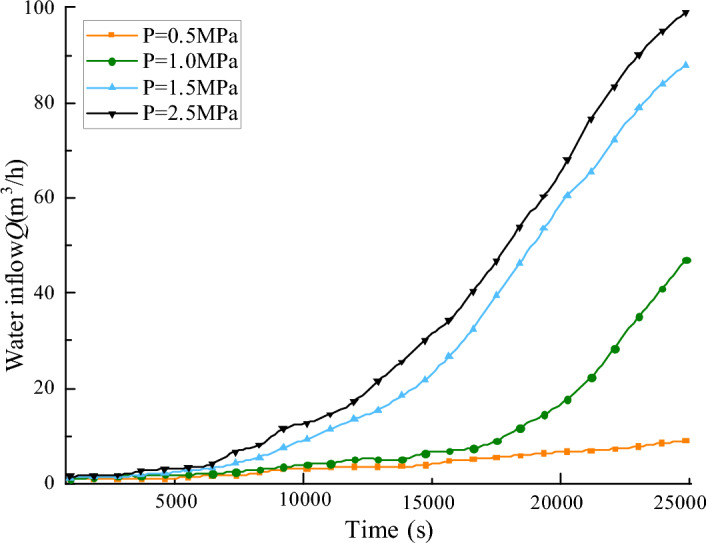
Influence of different water pressure on water inflow.

Figure 9 illustrates the curves depicting the relationship between water influx and time across different aquifer water pressures. It is evident from the Fig. 9 that higher aquifer water pressures result in greater water influx; the more pronounced the water channel is formed, the larger the porosity is, and the greater the increase at the outlet, resulting in a higher risk of water inrush. Initially, the increase in water inrush is relatively slow. As time progresses, at the moment of 15,000 s, the water inrush suddenly increases, which can be the trigger point for water inrush. This moment can be referred to as the “critical point of water inrush”.

#### Evolution of seepage velocity over time under different water pressures

**Figure 10:**
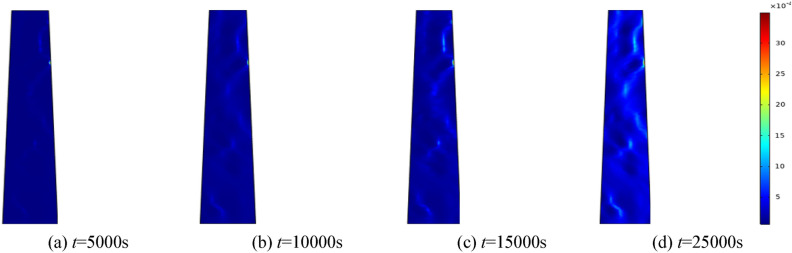
When the water pressure *p* = 0.5 MPa, seepage velocity distribution at different time.

**Figure 11:**
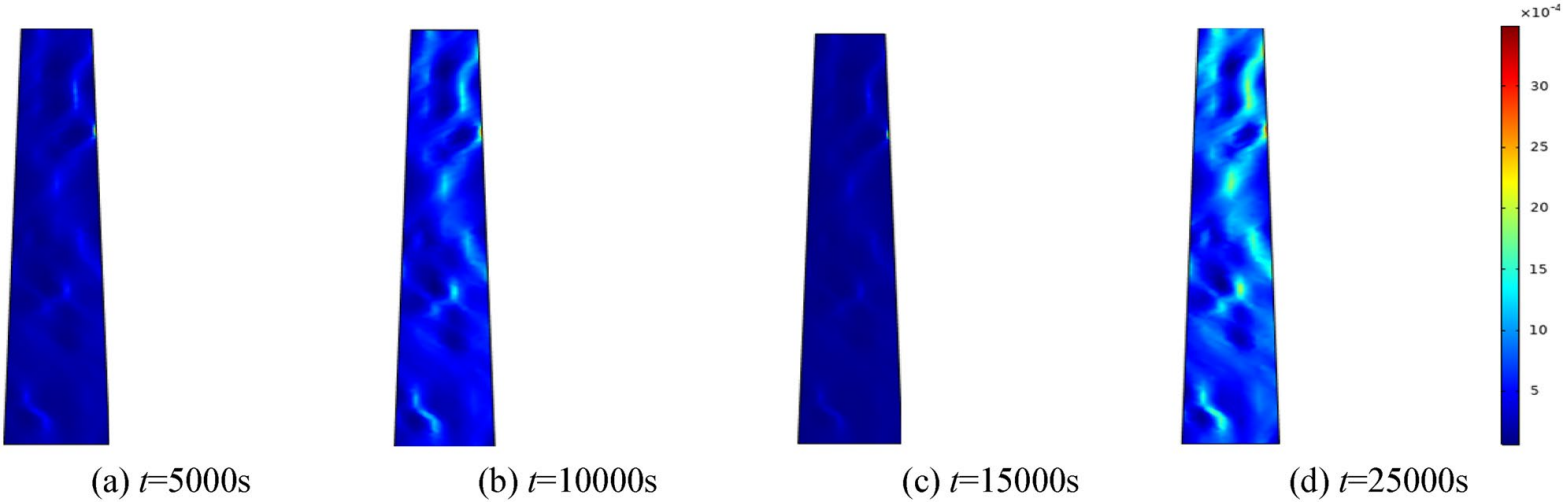
When the water pressure *p* = 1 MPa, seepage velocity distribution at different time.

**Figure 12:**
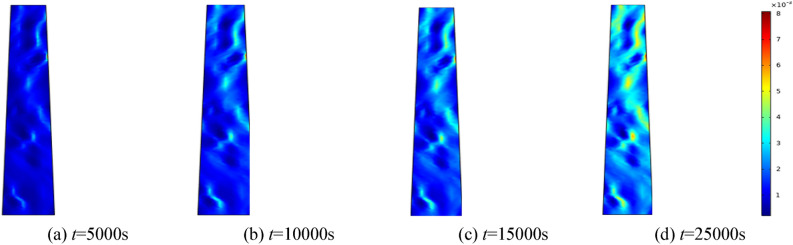
When the water pressure *p* = 1.5 MPa, seepage velocity distribution at different time.

**Figure 13:**
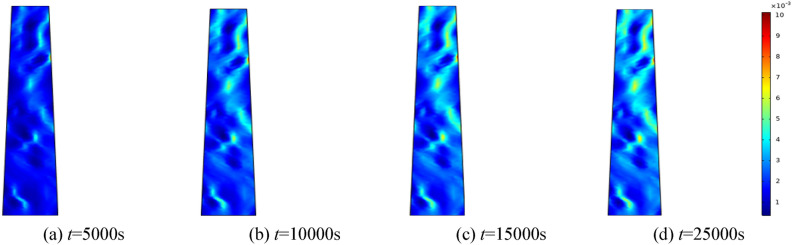
When the water pressure *p* = 1.5 MPa, seepage velocity distribution at different time.

To assess the impact of water pressure on the seepage rate of the crushed rock mass, water pressures of 0.5 MPa, 1.0 MPa, 1.5 MPa, and 2.0 MPa are examined. The homogeneity parameter is set at *m* = 3, and the surrounding rock of the fractured rock mass is maintained at 0.5 MPa. Selecting 5000 s, 10,000 s, 15,000 s, and 25,000 s for analysis. Figures 10, 11, 12, 13 show the distribution of the modeled seepage velocity at different time intervals for water pressures of 0.5 MPa, 1 MPa, 1.5 MPa and 2 MPa, respectively.

By analyzing the seepage velocity under various water pressures, it is evident that the growth rate of seepage velocity increases with increasing water pressure at the same time. Seepage velocity correlates with changes in porosity. As porosity enlarges, infiltration velocity intensifies. As the porosity change rate increases, the seepage velocity increases again. As seepage time increases, broken rock particles gradually migrate, undermining the internal pore and skeleton structure. Fine particles are relocated and eroded by water flow, modifying the seepage characteristics of the broken rock and establishing potential water-conducting channels. This leads to a significant augmentation in seepage rate. Eventually, the pores interconnect and form the primary channel of seepage flow.

### Effect of initial porosity on permeability properties of collapse column

#### Porosity evolution pattern for various initial porosities

**Figure 14:**
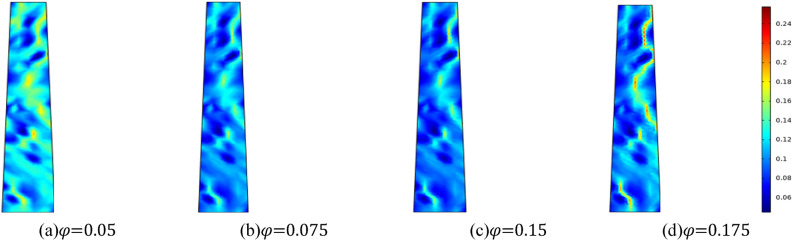
Different initial porosity, porosity distribution cloud map at the same time.

**Figure 15:**
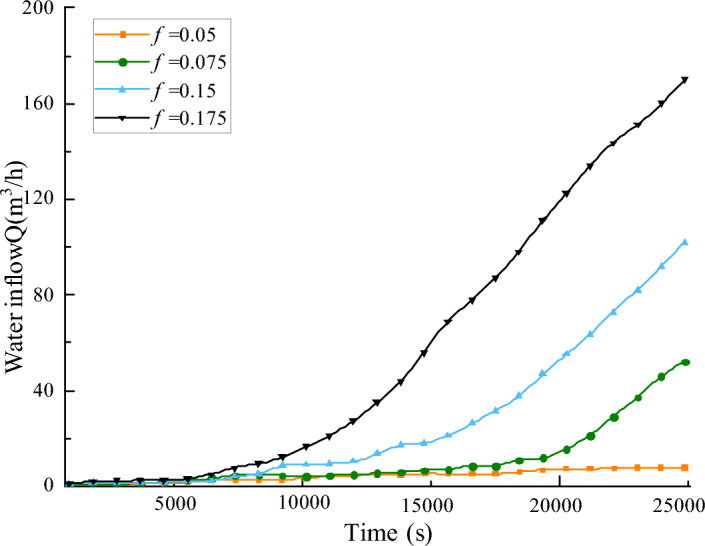
Relation curve of water inflow and time in broken rock mass with different initial porosity.

This paper investigates the evolution of permeability in fractured rock bodies with varying initial porosities of *φ* = 0.05, *φ* = 0.075, *φ* = 0.15, and *φ* = 0.175. In numerical simulation, the homogeneity parameter *m* is set to 3, the surrounding rock of the fractured rock mass is 0.5 MPa, and the applied water pressure is 2 MPa. Figure 14 illustrates the evolution of porosity at *t* = 25,000 s for fractured rock masses with varying initial porosities; Fig. 15 presents the relationship curve of water inflow with time under different initial porosity of the fractured rock masses. Figures 14 and 15 demonstrate that a larger initial porosity of the fractured rock mass results in a more pronounced water-conducting channel and a significant increase in porosity. The larger the initial porosity is, the more rapid the increase in water influx of the fractured rock mass is, which escalates the risk of a water inrush accident upon reaching the “critical point of time of water inrush”.

#### Evolution of Seepage Velocity with Varying Initial Porosity

**Figure 16:**
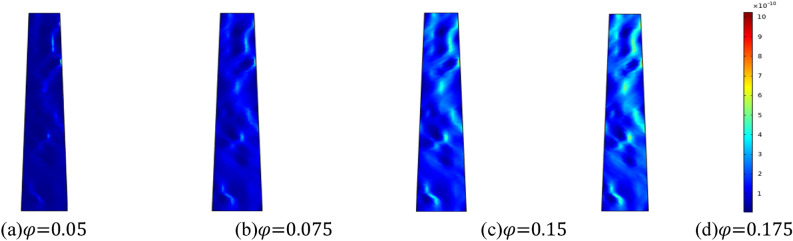
Distribution nephogram of seepage velocity at the same time with different initial porosity.

Considering the effect of initial porosity on the permeability of crushed rock mass, the initial porosity is set at 0.05, 0.075, 0.15 and 0.175, respectively. For the simulation, taking the homogeneity *m* = 3. Figure 16 shows the evolution characteristics of seepage velocity in fractured rock masses with different initial porosity when the water pressure in the aquifer is 2 MPa. Figure 16 illustrates that the seepage rate increases with the initial porosity. Water seepage and erosion prompt the fissures to progressively expand and coalesce, resulting in a more pronounced water channel, accelerating the seepage and flow of water, and leading to a sharp increase in water inrush, thereby increasing the risk of water inrush accidents.

#### Permeability evolution law under different initial porosity

**Figure 17:**
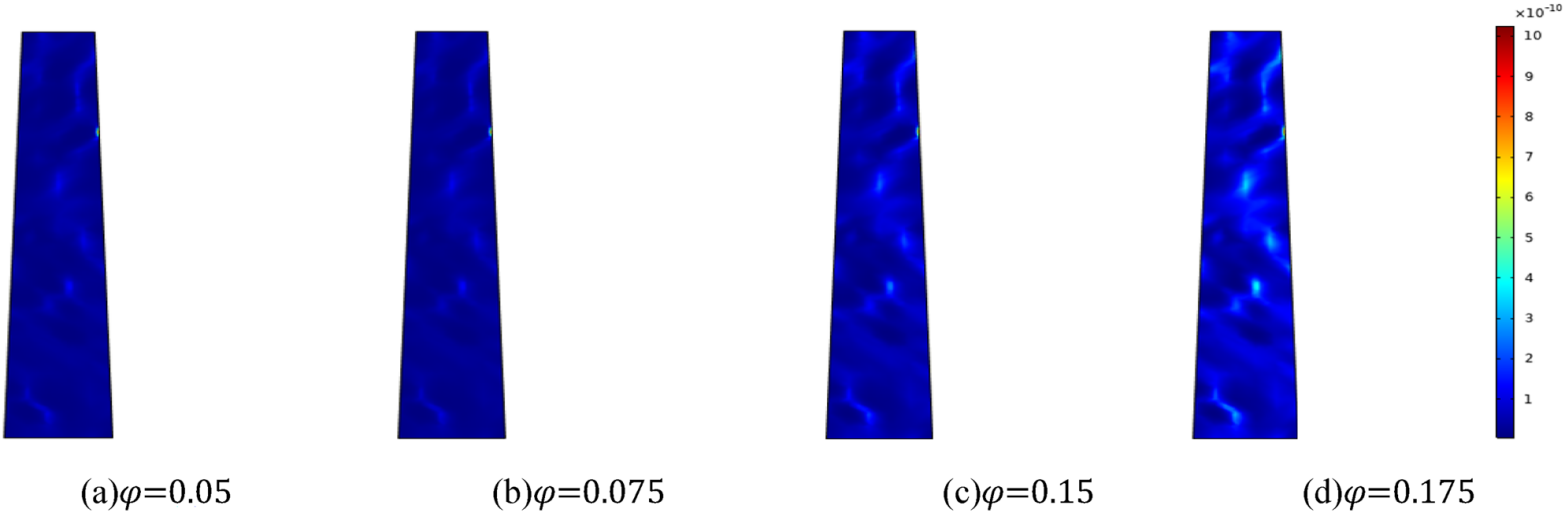
Distribution nephogram of permeability distribution at the same time with different initial porosity.

Figure 17 illustrates the evolution characteristics of permeability of fractured rock masses with varying initial porosity when the water pressure of the aquifer is 2 MPa and the homogeneity is *m* = 3. The Fig. 17 demonstrates that a larger initial porosity correlates with increased permeability. Water seepage and erosion induce the migration of mass particles, leading to an increase in permeability of the collapse column. As seepage channels develop, there is a loss of rock mass, culminating in a gradual increase in permeability. The changes in permeability and porosity exhibit a parallel trend.

## On-site monitoring of water inrush pattern of collapse column in working face

**Figure 18:**
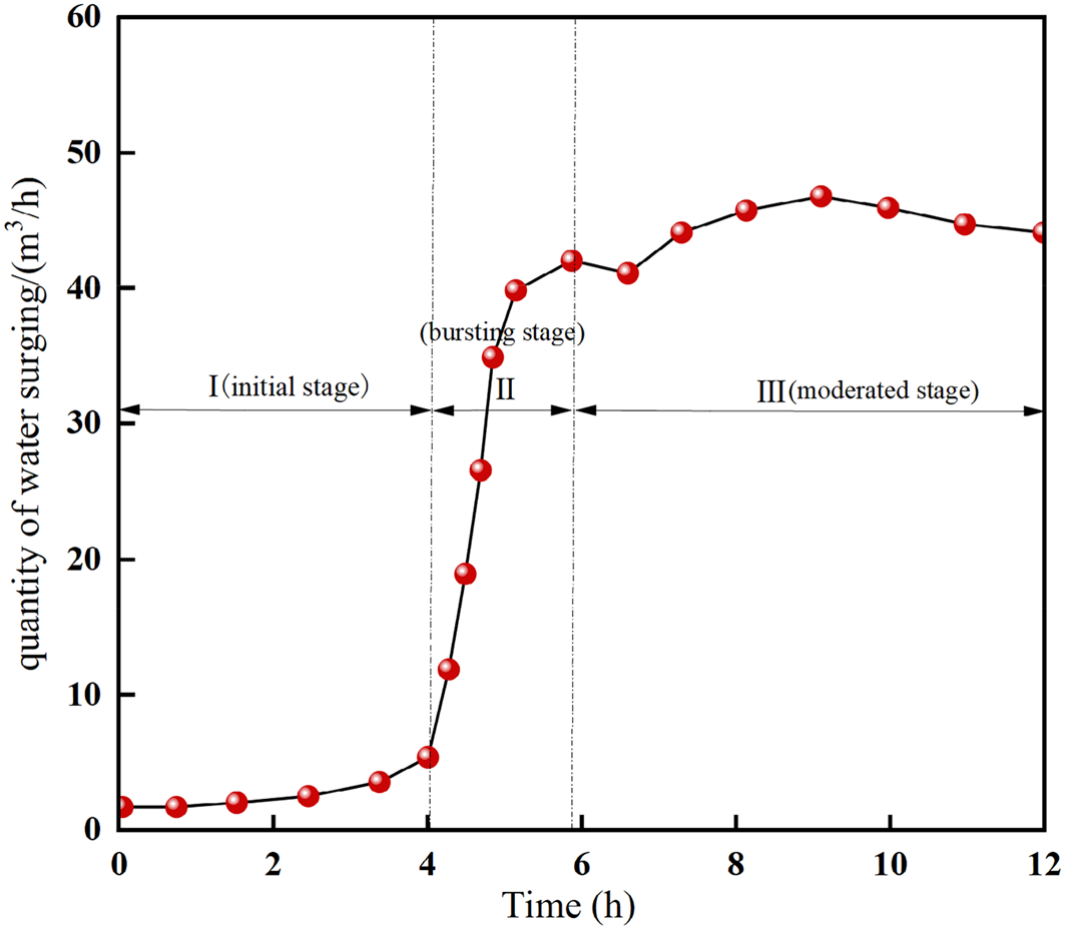
Dynamic detection curve of water quantity of collapse column in 1908 working face.

During the advancement of the working face passing through collapse column, real-time monitoring is carried out for the water inrush of collapse column at the 1908 working face of Qianjin coal mine. The dynamic monitoring curve of water-surging volume of the collapse column is shown in Fig. 18.

The monitoring curve reveals that the initial water inflow of the water-gushing collapse column is relatively low, escalating from 2.7 m^3^/h at the outset to 6.8 m^3^/h by the 4.2 h, with the water inflow changes slowly. However, the change speed of water inflow after 4.2 h obviously accelerates, with the water inflow escalating from 6.8 m^3^/h to 42.2 m^3^/h between 4.2 h and 5.9 h. The data indicate that the water inflow of the collapse column increases in vain after 4.2 h, and serious water inrush accident occurs, namely, this moment is the “critical point of water inrush” of the collapse column. If the measurement technology such as grouting reinforcement is taken before this time, the occurrence of water inrush accident can be effectively prevented.

By comparing the simulation and the field measurement data, the change trend of both is basically the same, which proves the correctness of variable mass fluid–solid coupling mechanical model of the collapse column established in this paper. The permeability change law of the fractured rock mass under the effect of the migration of filler particles is mastered, which is mainly divided into slow change stage, sudden change stage and stable stage. In the slow stage, the fracture opening degree and the water inflow inside the collapse column are small. Under the effect of water erosion, the particles of the rock mass particles migrate, the internal pores and skeletal structure are damaged, the permeability of the collapse column continues to increase, and some cracks continue to widen and penetrate to form potential water channels, resulting in a sharp increase in water inflow and eventually water collapse accidents.

## Conclusions

To solve the problem of water inrush of collapse column influenced by variable mass effect, a mechanical model of variety mass flow-solid coupling in the collapse column is established considering the random distribution characteristics of the collapse column. The seepage characteristics and the change law of water flow in the collapse column under different uniformity, water pressure, and initial porosity conditions are investigated by numerical simulation. The simulation results show that the change law of water inflow in the collapse column is consistent with the field measured data, which verifies the correctness of the variable mass flow-solid coupling mechanical model, and systematically explains the seepage law of variety mass and the occurrence mechanism of water inrush disaster in collapse column. The main conclusions are as follows:

1. Considering the influence of different homogeneity on the seepage of fractured rock masses, the higher the homogeneity is, the more water channels are formed and the higher the porosity value is. At the moment of 15,000 s, due to the continuous transport and loss of the filling material inside the collapse column, it leads to the expansion and interconnection of some fractures in the collapse column, and eventually forms a dominant water-conducting channel. After that, the water inflow of the collapse column suddenly increases, and a sudden water inrush accident occurs. This moment is the “critical point of water inflow” of the collapse column. If grouting reinforcement can be applied to the collapse column before this moment, it can prevent the occurrence of water inrush accidents.
2. When studying the evolution of seepage under different water pressures on fractured rock bodies, it is observed that higher water pressures lead to a rapid increase in porosity and seepage rate. As the seepage time increases, rock particles gradually migrate, causing damage to the internal pore and skeletal structure. Fine particles migrate and erode under the water flow, altering the seepage characteristics of the rock mass and potentially forming water-conducting channels.
3. When examining the effects of varying initial porosity on the seepage characteristics of a fractured rock masses, it is observed that higher initial porosity leads to increased permeability in the collapse column due to the migration of mass particles under the influence of water seepage and erosion. This results in the formation of more pronounced water-conducting channels and a faster attainment of the “critical moment points of water inrush”, consequently heightening the risk of water inrush.

## Data Availability

All data generated or analyzed during this study are included in this article.
